# RNF135 Promotes Human Osteosarcoma Cell Growth and Inhibits Apoptosis by Upregulating the PI3K/AKT Pathway

**DOI:** 10.1002/cnr2.2159

**Published:** 2024-08-08

**Authors:** Bingyao Chen, Yinglong Zhang, Guangze Song, Xing Wei

**Affiliations:** ^1^ Second Department of Orthopedics Beijing Daxing District People's Hospital Beijing China; ^2^ Department of Orthopedics Aerospace Central Hospital Beijing China; ^3^ Senior Department of Orthopedics The Fourth Medical Center of PLA General Hospital Beijing China

**Keywords:** apoptosis, osteosarcoma, PI3K/AKT, proliferation, RNF135

## Abstract

**Background:**

Ring finger protein 135 (RNF135) is an E3 ubiquitin ligase that has been implicated in the tumorigenesis of multiple human malignancies. However, whether RNF135 plays a role in the development of human osteosarcoma (OS) remains unknown.

**Methods:**

RNF135 expression in 20 human OS and 20 human osteochondroma specimens were evaluated by means of immunohistochemistry staining. The effects of shRNA‐mediated RNF135 knockdown on human OS cell growth and apoptosis were evaluated through a panel of in vitro studies on cell proliferation, colony formation, exposure of phosphatidylserine on the cell surface, and caspase 3/7 activation. The protein levels of PI3K, AKT, and p‐AKT were determined by western blot analysis.

**Results:**

We detected significantly higher RNF135 levels in human OS tissues than human osteochondroma tissues. In in vitro studies, shRNA‐mediated RNF135 knockdown in human OS cells inhibited proliferation and induced apoptosis. In addition, RNF135 knockdown reduced PI3K and p‐AKT protein levels and activated caspase 3 and 7.

**Conclusions:**

These results supported that RNF135 contributes to human OS development through PI3K/AKT‐dependent mechanisms. Targeting RNF135 may provide a new therapeutic approach for treating this human malignancy.

## 1 Introduction

Human osteosarcoma (OS) is a common, highly malignant bone tumor that mostly occurs in children and adolescents. It is characterized by rapid progression, early lung metastasis, and frequent postoperative recurrence. As such, OS patients suffer a high risk of morbidity and mortality, especially those with metastasis or recurrence [1]. Thanks to decades of advances in surgical techniques and combination chemotherapy, its 5‐year survival rate has increased from below 20% to about 70% in the absence of metastases and 30% if metastases are present at diagnosis [2, 3]. The emergence of immunotherapy as an effective treatment for certain cancers has prompted the evaluation of immune checkpoint inhibitors as potential therapies for OS. Nonetheless, recent clinical trials have shown limited improvement over conventional treatment modalities [4]. In addition, treatment strategies specifically targeting AURKB, CCNE1/CDK2, CDK4/CDK6/FOXM1, MYC/CDK9, PTEN/PI3K/AKT1/mTOR, and VEGFA/VEGFR have been evaluated in OS patients [5]. However, current targeted therapy has not brought significant improvement in patient clinical outcome.

Protein ubiquitination is a tightly regulated, highly specific posttranslational modification that regulates protein activation, localization, and degradation. It is involved in almost all aspects of eukaryotic biology [6]. In a three‐enzyme ubiquitination cascade, E3 ubiquitin ligases work together with E1 ubiquitin activating enzymes and E2 ubiquitin conjugating enzymes to catalyze the ubiquitination process and transfer and attach ubiquitin to specific lysine residues of targeted proteins. Dysregulation of the ubiquitination process may lead to carcinogenesis [7]. Notably, E3 ligases are recognized as the second most common functional family associated with carcinogenesis [8]. Really interesting new gene (RING) E3 ligases, the major type of E3 ligases, are frequently upregulated in various tumors and are considered potential therapeutic targets for cancer [9, 10].

RING finger protein 135 (RNF135), a RING E3 ligase, was first identified by Oshiumi et al. as a novel component of the RIG‐I pathway responsible for type I interferon induction during early immune response to RNA virus infection [11]. RNF135 contains a N‐terminal RING domain and C‐terminal SPRY and PRY motifs, and both the RING domain and the SPRY and PRY motifs are important for its biological function. Subsequent studies have linked RNF135 to carcinogenesis. In a pan‐cancer analysis, RNF135 was found to be dysregulated in many human cancers, and its expression level correlated with disease progression and prognosis [12]. In addition, various studies have shown that RNF135 plays a significant role in the proliferation and dissemination of multiple cancers such as tongue cancer [13], glioblastoma (GBM) [14, 15], hepatocellular carcinoma [16], and breast cancer [12]. However, RNF135 expression and function in human OS remain unknown.

In this study, we evaluated the expression of RNF135 in human OS tissues and investigated the effects of short hairpin RNA (shRNA)‐mediated RNF135 knockdown on human OS cell growth and apoptosis. The possible molecular mechanisms by which RNF135 influences human OS were also investigated.

## 2 Methods

### 2.1 Patients and Specimens

Tumor specimens were collected from 20 OS patients who were admitted to the Fourth Medical Center of PLA General Hospital (Beijing, China) between 2012 and 2016. Osteochondroma specimens collected from 20 osteochondroma patients admitted during the same period served as control. We used osteochondroma specimens as control because osteochondroma is the most common benign bone tumor, and it resembles OS in terms of age of onset and tumor origin and anatomic locations [17, 18]. The 20 OS patients included 14 males and 6 females aged 6–34, with a median age of 16.2 years. Eight patients had the disease in the distal femur, nine in the proximal tibia, and three in other bone locations. The 20 osteochondroma patients included 12 males and 8 females aged 7–41, with a median age of 19.6 years. Patients who received radiotherapy or chemotherapy before surgery were excluded. The study protocol was approved by the Ethics Committee of PLA General Hospital. All participants gave informed consent.

### 2.2 Immunohistochemistry

RNF135 was detected using immunohistochemistry (IHC) staining with an anti‐RNF135 antibody (1:100; Proteintech, USA, Cat# 25061‐1‐AP) and an IHC Kit (Zhongshan Jinqiao Biotechnology, Beijing, China, Cat# ZK‐9600). Sample preparation and IHC staining were conducted by a senior pathologist. After staining, the slides were examined and graded by two highly trained pathologists as previously described [17]. The staining was categorized into four levels as follows: negative (−), weak (+), moderate (++), and strong (+++).

### 2.3 Cell Culture

The U2OS, Saos‐2, MG‐63, and HOS OS cell lines were obtained from National Collection of Authenticated Cell Cultures (Shanghai, China). U2OS and Saos‐2 human OS cells were grown in McCoy's 5A (modified) Medium (Hyclone, USA, Cat# SH30200.01) containing 15% and 10% fetal bovine serum (FBS, Hyclone, Cat# SV30208.03), respectively. MG‐63 and HOS human OS cells were grown in Eagle's Minimum Essential Medium (Hyclone, Cat# SH30024.01) containing 10% FBS. All cell cultures were maintained at 37°C in a humidified atmosphere containing 5% CO_2_.

### 2.4 Quantitative Reverse Transcription PCR

To determine RNF135 mRNA expression, total RNA was extracted using Trizol reagent (Pufei, China, Cat# 3101‐100) and sent to Sangon Biotech Company (China) for quantitative reverse transcription PCR (qRT‐PCR) analysis. The primer sequences for human RNF135 were 5′‐GGAGCTGTGAGAGGTTTTCTAC‐3′ (forward) and 5′‐CATTCCACACAACAAGAGTCC‐3′ (reverse). The primer sequences for human GAPDH were 5′‐TGACTTCAACAGCGACACCCA‐3′ (forward) and 5′‐CACCCTGTTGCTGTAGCCAAA‐3′ (reverse). The 2^−ΔΔCt^ method was employed to determine RNF135 mRNA levels relative to GAPDH.

### 2.5 Western Blot Analysis

The cells were lysed in RIPA lysis buffer in the presence of protease and phosphatase inhibitors (Roche, Cat# 04693159001 and 04906845001). The lysates were centrifuged at 12000 rpm for 20 min. The proteins in the supernatants (50 μg of each sample) were separated by SDS‐PAGE, transferred to PVDF membranes, and probed with antibodies toward RNF135 (1:1000; Proteintech, USA, Cat# 25061‐1‐AP), AKT (1:3000; Cell Signaling Technology, USA, Cat# 4691S), p‐AKT (1:1000; Proteintech, USA, Cat# 66444‐1‐Ig), PI3K (1:1000, Cell Signaling Technology, USA, Cat# 4249), and GAPDH (1:30000; Proteintech, USA, Cat# 60004‐1‐Ig), respectively. After incubation with horseradish peroxidase (HRP)‐conjugated secondary antibodies (1:3000; Beyotime, China, Cat# A0208 and A0216), the protein bands were detected with Immobilon HRP Substrate (Millipore, USA, Cat# RPN2232) on a Bio‐Rad gel imager. Data were normalized to GAPDH.

### 2.6 ShRNA‐Mediated RNF135 Knockdown

Lentiviral vectors carrying a shRNA targeting human RNF135 (shRNF135‐1, ‐2, or ‐3) or a negative control shRNA (shNC) were obtained from Shanghai Genechem (Shanghai, China). The RNF135 knockdown efficiencies in HOS cells were evaluated with qRT‐PCR as well as western blot analysis. The shRNF135 showing the greatest knockdown efficiency in HOS cells was used for RNF135 knockdown in U2OS cells. Stable RNF135 knockdown U2OS and HOS cells and their respective controls were generated and used for subsequent experiments.

### 2.7 Cell Proliferation Assay (Celigo Cell Counting Assay)

Stable RNF135 knockdown HOS and U2OS cells and their respective controls were seeded into 96‐well plates (2 × 10^3^ cells per well, day 0). The 96‐well plates were scanned on a Celigo imaging cytometer (Nexcelom, USA) once daily from day 1 to day 5 to obtain cell counts, which were used to create a cell proliferation curve.

### 2.8 Colony Formation Assay

Stable RNF135 knockdown HOS and U2OS cells and their respective controls were seeded into 6‐well plates (800 cells per well) and incubated for 15 days. After washing with PBS, the cells were fixed in 4% polyoxymethylene and stained with 4% crystal violet. The cell colonies were counted and photographed.

### 2.9 Annexin V‐APC Apoptosis Assay

Stable RNF135 knockdown HOS and U2OS cells and their respective controls were seeded into 6‐well plates (8 × 10^5^ cells per well). After a 24‐h incubation, the cells were trypsinized and collected by centrifugation at 1300 rpm for 5 min. The cell pellets were washed and placed in precooled D‐Hanks buffer, and apoptosis was determined on a FACSCalibur flow cytometer (BD Biosciences, USA) using the eBioscience Annexin V Apoptosis Detection Kit APC (Invitrogen, USA, Cat# 88‐8007). The percentage of apoptotic cells was calculated using CellQuest version 3.3 (BD Biosciences).

### 2.10 Caspase Assay

Caspase 3/7 activities were determined using the Caspase‐Glo 3/7 Assay Kit (Promega, USA, Cat# G8091). The luminescent signals were recorded on a microplate reader.

### 2.11 Statistical Analysis

All data are presented as the means ± standard deviation (SD). All data analyses were conducted using the SPSS 12.0 Software. The IHC data were compared using the *χ* ^2^ test with Yates' correction. All other data were compared using a two‐sided Student's *t*‐test or one‐way analysis of variance (ANOVA). A *p* value less than 0.05 was deemed statistically significant.

## 3 Results

### 3.1 RNF135 is Upregulated in Human OS Tissues

**FIGURE 1:**
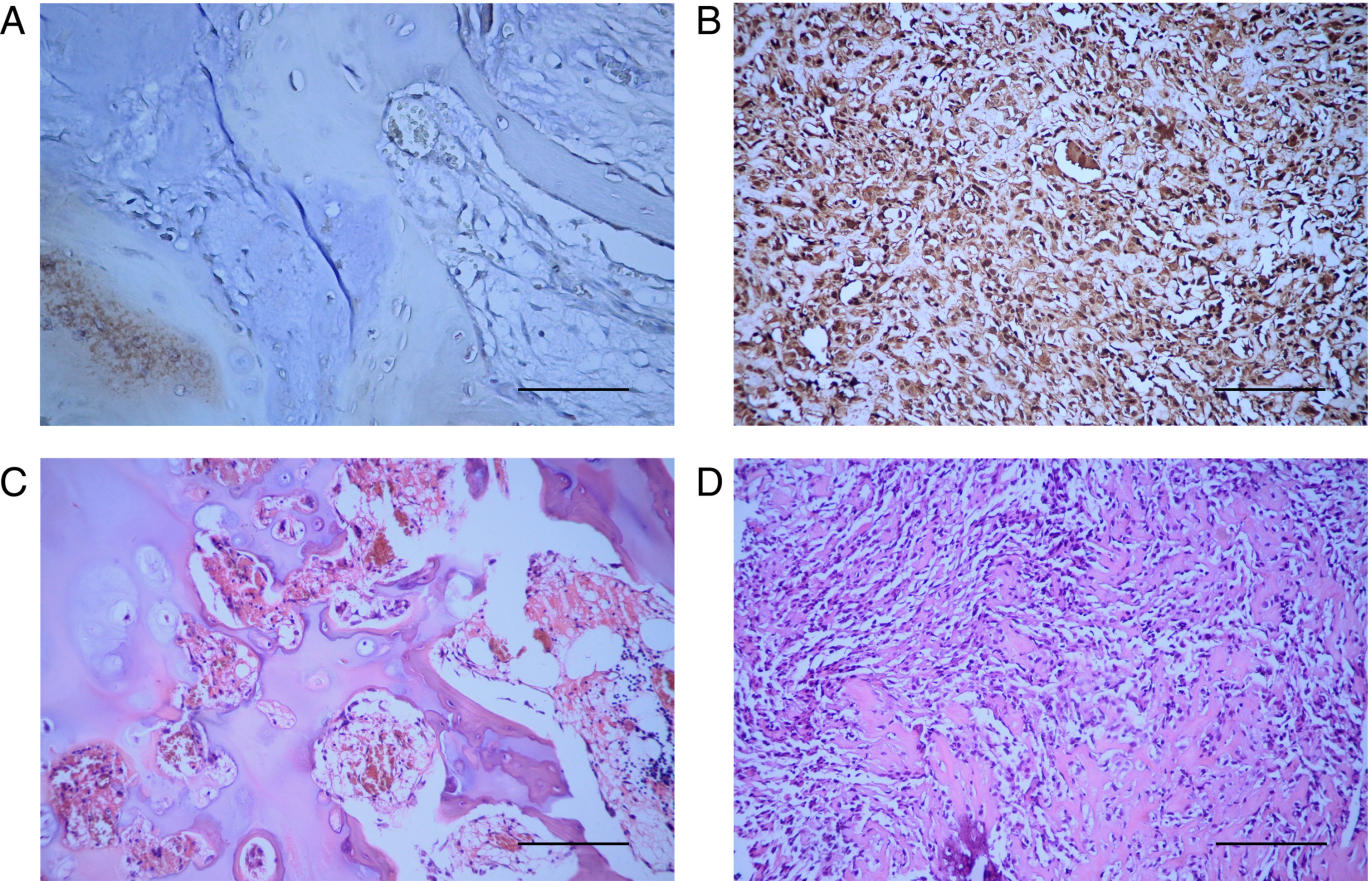
RNF135 is upregulated in human OS tissues. (A, B) Representative images of IHC staining for RNF135 in human osteochondroma (A) and OS tissues (B) at 400× magnification. Both tissues were counterstained with hematoxylin. (C, D) Representative images of H&E staining of human osteochondroma (C) and OS tissues (D) at 200× magnification. (*n* = 20, Scale bar = 200 μm).

IHC and H&E staining of human OS and osteochondroma tissues revealed that RNF135 was mainly localized in the cell membrane and cytoplasm, with OS tissues showing markedly higher staining signal than osteochondroma tissues (Figure 1A–D). Semiquantitative immunoreactivity evaluation showed that 90% of the cell population in OS tissues were RNF135 positive while only 15% were positive in osteochondroma tissues (Table 1, *p* < 0.001). These data indicated that RNF135 is upregulated in human OS.

**TABLE 1:** IHC staining for RNF135 in human osteochondroma and OS tissues.

Tissue	*n*	−	+	++	+++	Percent positive (%)
Osteochondroma	20	17	3	0	0	15
Osteosarcoma	20	2	5	6	7	90***

*Note*: −, negative; +, weak; ++, moderate; and +++, strong.

*** *p* < 0.001.

### 3.2 RNF135 is Expressed in Human OS Cell Lines

**FIGURE 2:**
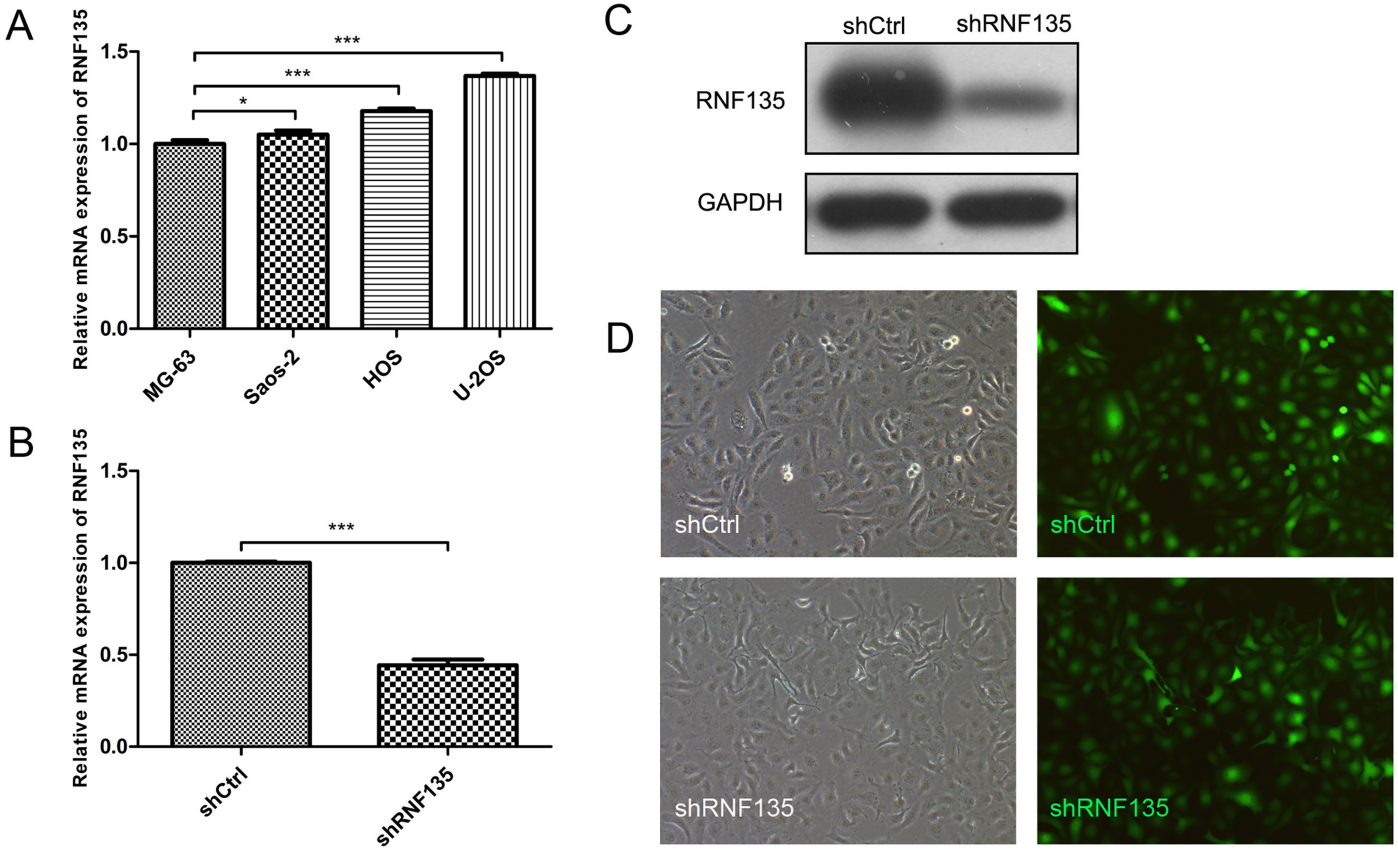
RNF135 expression in human osteosarcoma cell lines and shRNA‐mediated RNF135 knockdown in U2OS cells. (A) RNF135 mRNA expression in MG‐63, Saos‐2, HOS, and U2OS human osteosarcoma cell lines by qRT‐PCR. *n* = 3, *p < 0.05, ***p < 0.001. (B, C) U2OS cells were transfected with shRNF135‐1 or shNC. RNF135 mRNA (B) and protein (C) levels were determined by qRT‐PCR and western blot analysis, respectively. *n* = 3, ***p < 0.001. (D) U2OS cells were transfected with shRNF135‐1 or shNC. Representative bright‐field (left panels) and fluorescence (right panels) cell images are shown.

Next, we evaluated the mRNA expressions of RNF135 in human OS cell lines including MG‐63, Saos‐2, HOS, and U2OS using qRT‐PCR. As shown in Figure 2A, RNF135 expression followed the order of U2OS > HOS > Saos‐2 > MG‐63. We selected U2OS and HOS cells, which showed the highest and second highest RNF135 expression of the four cell lines, for subsequent RNF135 knockdown experiments to investigate the role of RNF135 in human OS cell growth and apoptosis.

### 3.3 RNF135 Knockdown Inhibits Human OS Cell Growth and Colony Formation

**FIGURE 3:**
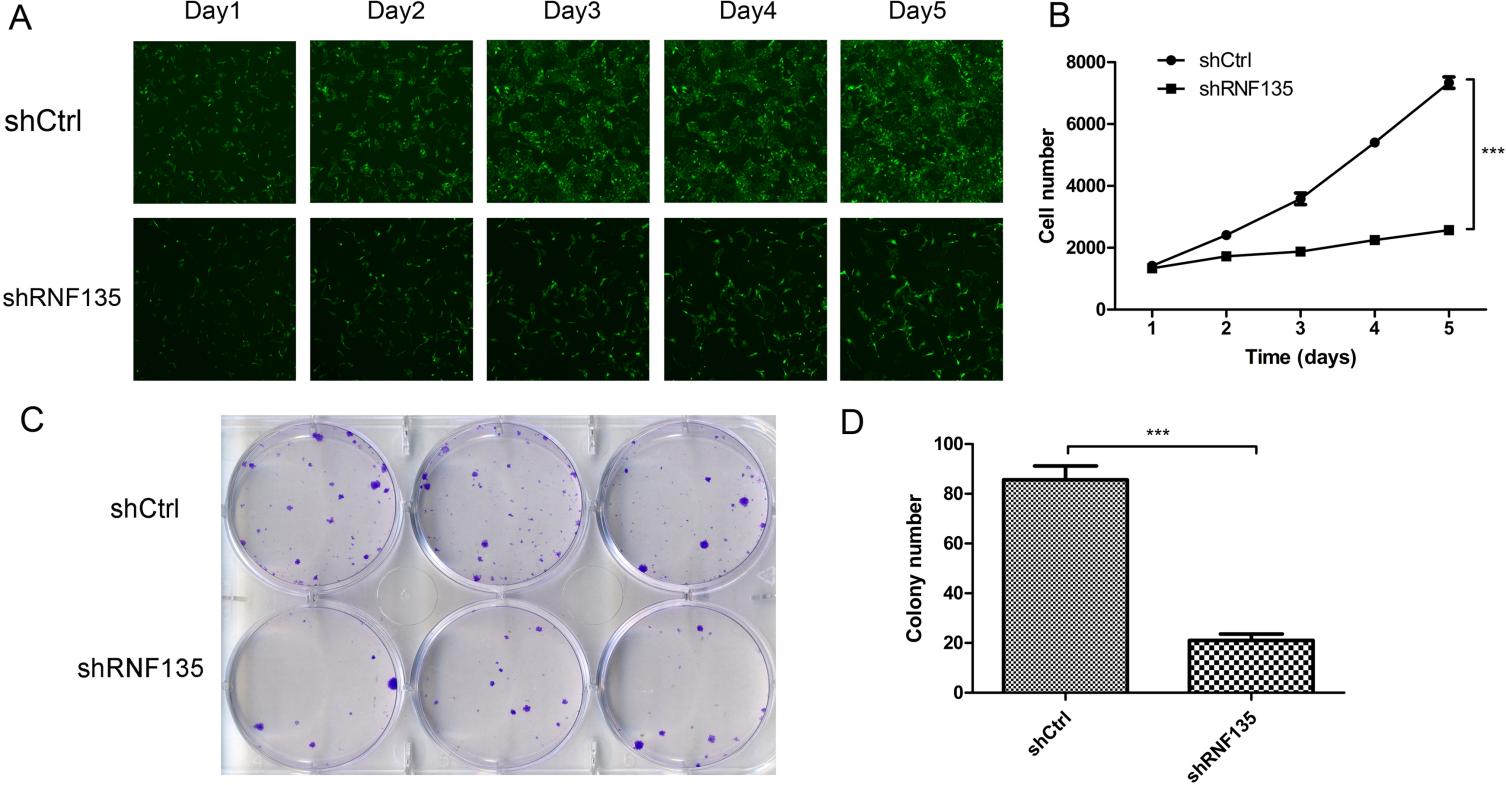
RNF135 knockdown inhibits U2OS cell proliferation and colony formation. (A, B) Stable RNF135 knockdown and shNC control U2OS cells were cultured in 96‐well plates for 5 days. Representative fluorescence cell images (A) and cell growth curve (B) are shown. *n* = 3, ***p < 0.001. (C, D) Stable RNF135 knockdown and shNC control U2OS cells were cultured in 6‐wells plates for 15 days. Representative cell images (C) and the number of colonies formed (D) on day 15 are shown. *n* = 3, ***p < 0.001.

HOS cells were transfected with shRNF135‐1, ‐2, ‐3, and shNC, respectively. qRT‐PCR and western blot analysis showed that all three shRNF135s downregulated RNF135 expression, with shRNF135‐1 exhibiting the greatest silencing efficiency (Figure S1A,B). Therefore, shRNF135‐1 was used for RNF135 knockdown in U2OS cells (Figure 2B,C). Fluorescence imaging revealed a greater than 80% shRNA transfection efficiency (Figure 2D). To evaluate the effects of RNF135 knockdown on human OS cell growth, stable RNF135 knockdown and shNC control U2OS cells were cultured for 5 days in 96‐well plates. Cell proliferation at days 1–5 was evaluated under a microscope and quantitatively determined on a Celigo imaging cytometer. Compared with control cells, the RNF135 knockdown cells exhibited a significantly slower growth rate (Figure 3A,B). In addition, after 15 days of incubation in 6‐well plates, the knockdown cells formed fewer colonies than control (Figure 3C,D). Similarly, RNF135 knockdown in HOS cells inhibited cell proliferation and colony formation (Figure S1C–F). Together, these data demonstrated the proliferative function of RNF135 in human OS cells.

### 3.4 RNF135 Knockdown Activates Caspase 3/7 and Induces Human OS Cell Apoptosis

**FIGURE 4:**
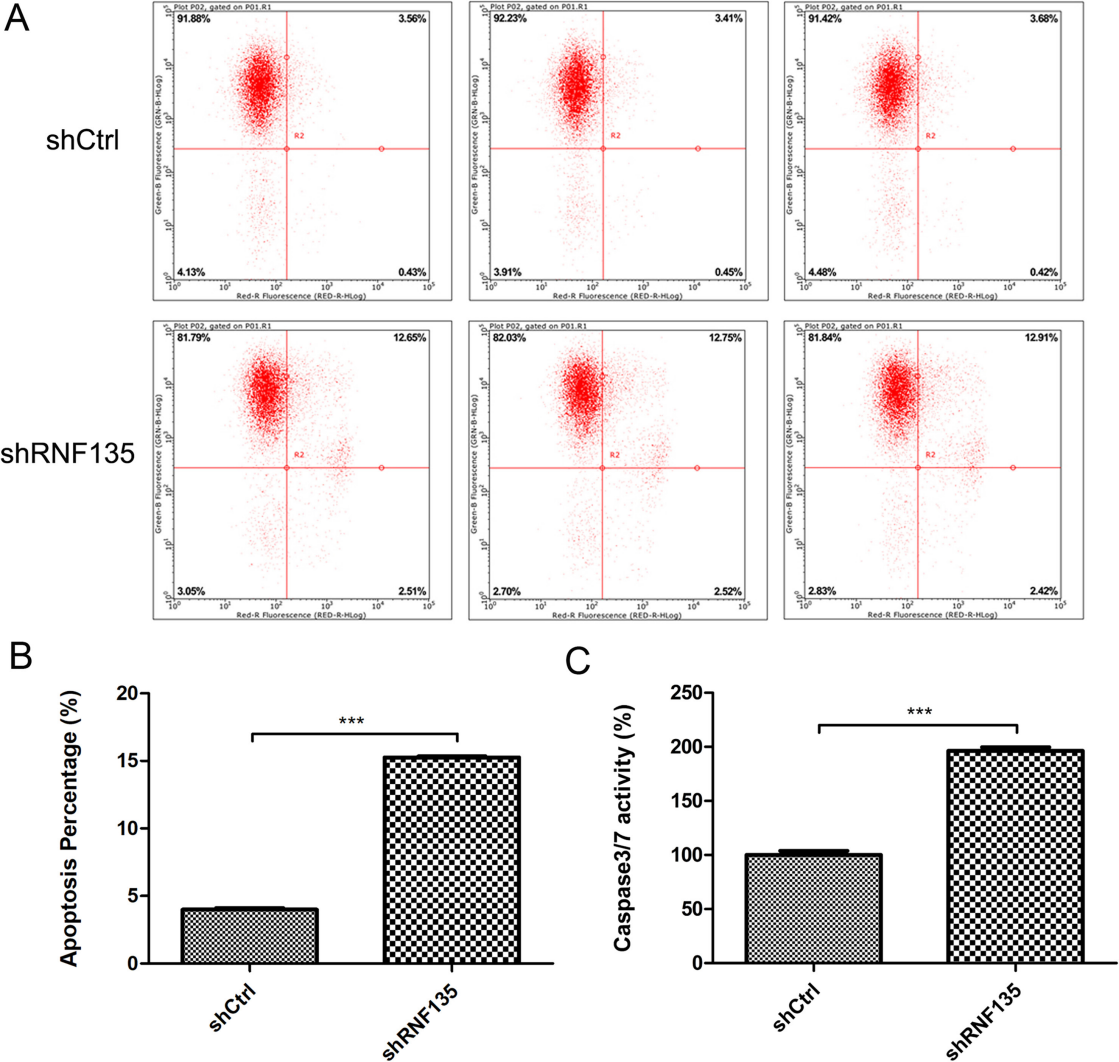
RNF135 knockdown activates caspase 3/7 and induces U2OS cell apoptosis. Stable RNF135 knockdown and shNC control U2OS cells were cultured in 6‐well plates for 24 h. (A, B) Flow cytometric analysis of apoptosis using Annexin V staining. Representative flow cytometry histograms (A) and quantified apoptotic cell percentages (B) are shown. *n* = 3, ***p < 0.001. (C) Caspase 3/7 activity determined by the luminescence Caspase‐Glo assay. *n* = 3, ***p < 0.001.

To evaluate the effects of RNF135 knockdown on human OS cell apoptosis, stable RNF135 knockdown and shNC control U2OS cells were cultured in 6‐well plates for 24 h. Apoptosis was detected by flow cytometry with Annexin V straining. The knockdown cells exhibited an apoptotic index of 15.5%, markedly greater than that of control cells, which was 3.9% (Figure 4A,B). Similarly, RNF135 knockdown increased apoptosis in HOS cells (Figure S2A,B). Compared with control cells, RNF135 knockdown U2OS cells showed significantly increased activity of caspase 3/7 (Figure 4C), which are critical mediators of mitochondrial apoptosis [19].

### 3.5 RNF135 Knockdown Downregulates PI3K/AKT in Human OS Cells

The PI3K/AKT signaling is a master regulator of apoptosis. To investigate the molecular mechanisms underlying the effects of RNF135 knockdown on apoptosis, we evaluated the protein levels of PI3K, AKT, and p‐AKT using western blot analysis. Although showing no effects on total AKT, NRF135 knockdown reduced PI3K, and p‐AKT protein expression in both U2OS and HOS cells (Figure S2C,D), indicating that NRF135 knockdown induces apoptosis at least partially by downregulating the PI3K/AKT pathway.

## 4 Discussion

In this study, we detected drastically higher RNF135 expression in human OS tissues than human osteochondroma tissues. ShRNA‐mediated RNF135 knockdown inhibited human OS cell growth and colony formation and promoted apoptosis. Mechanistically, RNF135 knockdown downregulated the PI3K/AKT pathway and activated caspase 3/7. These findings suggested that RNF135 contributes to human OS tumorigenesis by upregulating the PI3K/AKT pathway.

Although OS is a common primary bone malignancy in children and young adults, its etiology and molecular pathology have not been fully elucidated. Ubiquitination, a posttranslational modification that regulates protein function and degradation, has been closely linked to the development of OS in research over the past decades [20]. Many ubiquitination‐related enzymes have been implicated in human OS, and the majority of these enzymes are E3 ubiquitin ligases, especially RING E3 ubiquitin ligases [20]. For example, the oncoprotein MDM2 is a RING E3 ligase that ubiquitinates and antagonizes p53 to promote OS development [21, 22]. The TRIM family of proteins is defined by N‐terminal B‐box and coiled‐coil motifs and a RING finger region that has E3 ubiquitin ligase activity. Studies have shown that TRIM11 and TRIM46 are upregulated in human OS and can promote OS cell growth by activating the ERK1/2 and NF‐κB signaling pathways, respectively [23, 24]. TRIM7 is also upregulated in human OS and can enhance OS cell migration, invasion, and chemoresistance through ubiquitination of BRMS1 [25]. Cullin‐RING E3 ubiquitin ligase (CRL) is the largest E3 ligase family [26]. The CRL family member CRL4B has been shown to promote cell cycle progression and proliferation of human OS cells through ubiquitination of PTEN [27, 28]. Interestingly, while most OS‐related RING E3 ubiquitin ligases have been reported to be upregulated in tumor tissues and enhance OS development, TRIM58 and RNF180 were found to be downregulated in human OS and inhibit OS progression through ubiquitination of pyruvate kinase M2 and chromobox homolog 4, respectively [29, 30]. These findings indicate that RING E3 ubiquitin ligases regulate OS development through complex cellular and molecular mechanisms, and they can either promote or curb OS tumorigenesis. Pharmaceutical intervention of RING E3 ubiquitin ligases may provide a new therapeutic modality to treat human OS.

Since its identification in 2009 [11], RNF135 has been linked to many human cancers [12]. For example, Liu et al. found that RNF135 can activate the ERK pathway in human GBM cells to promote cell proliferation and migration, and RNF135 downregulation can curb human GBM xenograft growth in nude mice [15]. Beta‐elemene is a herb‐derived terpene that exhibits antiproliferative properties in many cancer cells through cell cycle arrest and apoptosis induction [31]. Alizada et al. discovered that beta‐elemene inhibits human GBM tumorigenesis in vitro and in vivo by downregulating RNF135 [14]. MiR‐485‐3p is a biomarker for poor prognosis in GBM patients [32]. Zhang et al. reported that miR‐485‐3p downregulates RNF135 to inhibit GBM cell proliferation and migration [33]. Together, these findings have demonstrated that RNF135 plays an oncogenic role in human GBM. In addition to GBM, RNF135 has been linked to increased risk of malignant peripheral nerve sheath tumors (MPNSTs) in neurofibroma patients with NF1 microdeletion [34]. Controversially, Jin et al. found that RNF135 can inhibit tongue cancer tumorigenesis in vitro and in vivo by upregulating PTEN and TP53 [13]. In addition, RNF135 is downregulated in human hepatocellular carcinoma (HCC) and its expression is positively associated with tumor infiltration of immune cells and patient survival. Also, RNF135 knockdown in HCC cells leads to increased cell migration [16]. Therefore, the current literature suggests that RNF135 may exhibit oncogenic or antitumor effects, depending on the specific type of tumor involved.

In this study, we evaluated RNF135 expression in 20 human OS and 20 human osteochondroma specimens by means of IHC staining. We detected RNF135 expression in 90% of the cell population in OS specimens, but only 15% of the cell population in osteochondroma specimens stained positive for RNF135, showing drastic RNF135 upregulation in human OS. In both OS and osteochondroma specimens, RNF135 protein was localized to the cytoplasm and cell membrane. Subsequent in vitro studies with shRNA‐mediated RNF135 knockdown demonstrated the proliferative and antiapoptotic function of RNF135 in U2OS and HOS human OS cells. As such, findings from this study supported that RNF135 plays an oncogenic role in human OS and hence, it may serve as a new therapeutic target for this hard‐to‐treat malignancy. Notably, the IHC results from this study revealed significant heterogeneity in RNF135 expression among human OS specimens. This heterogeneity may have resulted from genetic and epigenetic variations between individual patients [35, 36]. Future studies are required to investigate whether RNF135 expression is linked to clinical manifestations and prognosis in human OS.

Mechanistically, we identified the PI3K/AKT pathway and caspase 3/7 as downstream mediators of RNF135. The PI3K/AKT pathway is a very complex cell signaling pathway composed of numerous molecular components [37]. The detailed molecular mechanisms by which RNF135 upregulates this pathway in human OS awaits further investigation. Inhibitor of apoptosis proteins (IAPs) can function as E3 ubiquitin ligases that bind to and ubiquitinate caspases to inhibit cancer cell apoptosis [38]. Whether RNF135 inactivates caspase 3/7 through direct ubiquitination or indirect mechanisms, for example, through PI3K/AKT, remains to be investigated. Co‐localization immunofluorescence of RNF135, PI3K/AKT, and caspases in human OS cells, as well as immunoprecipitation and co‐immunoprecipitation studies may be performed in the future to answer these questions. Another limitation of this study was that the effects of RNF135 knockout were not investigated. Completely knocking‐out RNF135 in OS cells could further enhance caspase‐mediated apoptosis, leading to massive cell death. We may evaluate the effects of RNF135 knockout in human OS cells in future research. Also, studies utilizing in vivo xenograft models are required to confirm the oncogenic effects of RNF135 in human OS.

## 5 Conclusions

In summary, we found that RNF135 is upregulated in human OS tissues. RNF135 knockdown can inhibit human OS cell growth and colony formation and induce apoptosis by downregulating the PI3K/AKT pathway. Therefore, RNF135 contributes to human OS tumorigenesis and hence, it may serve as a new target for this malignant disease.

## Author Contributions

**Bingyao Chen:** funding acquisition (equal), methodology (lead), writing – original draft (lead). **Yinglong Zhang:** formal analysis (lead), funding acquisition (equal), software (lead), writing – review and editing (lead). **Guangze Song:** data curation (lead), project administration (lead), resources (lead). **Xing Wei:** conceptualization (lead), validation (lead).

## Ethics Statement

All the experiments and procedures were approved by both the Ethics Committee of The Fourth Medical Center of PLA General Hospital and the Ethics Committee of Aerospace Central Hospital (No.2022‐74).

## Conflicts of Interest

The authors declare no conflicts of interest.

## Supporting information


**Figure S1.** RNF135 knockdown inhibits HOS cell proliferation and colony formation. (A, B) HOS cells were transfected with shRNF135‐1, ‐2, ‐3, or shNC. RNF135 mRNA (A) and protein (B) levels were determined by qRT‐PCR and western blot analysis, respectively. *n* = 3, **p < 0.01, ***p < 0.001. (C, D) Stable RNF135 knockdown and shNC control HOS cells were cultured in 96‐well plates for 5 days. Representative fluorescence cell images (C) and cell growth curve (D) are shown. *n* = 3, ***p < 0.001. (E, F) Stable RNF135 knockdown and shNC control HOS cells were cultured in 6‐wells plates for 15 days. Representative cell images (E) and the number of colonies formed (F) on day 15 are shown. *n* = 3, **p < 0.01.

**Figure S2.** RNF135 knockdown downregulates PI3K/AKT and induces HOS cell apoptosis. Stable RNF135 knockdown HOS and U2OS cells and their respective shNC control cells were cultured in 6‐well plates for 24 h. (A, B) Flow cytometric analysis of HOS cell apoptosis using Annexin V staining. Representative flow cytometry histograms (A) and quantified apoptotic cell percentages (B) are shown. *n* = 3, ***p < 0.001. (C, D) The protein levels of AKT, p‐AKT, and PI3K in HOS (C) and U2OS (D) cells determined by western blot analysis.

## Data Availability

The data that support the findings of this study may be available upon reasonable request per approval from The Fourth Medical Center of PLA General Hospital and Aerospace Central Hospital authorities. Further information is available from the corresponding author upon request.

## References

[1] D. J. Harrison , D. S. Geller , J. D. Gill , V. O. Lewis , and R. Gorlick , “Current and Future Therapeutic Approaches for Osteosarcoma,” Expert Review of Anticancer Therapy 18, no. 1 (2018): 39–50. 29210294 10.1080/14737140.2018.1413939

[2] J. C. Friebele , J. Peck , X. Pan , M. Abdel‐Rasoul , and J. L. Mayerson , “Osteosarcoma: A Meta‐Analysis and Review of the Literature,” American Journal of Orthopedics 44, no. 12 (2015): 547–553. 26665241

[3] A. Luetke , P. A. Meyers , I. Lewis , and H. Juergens , “Osteosarcoma Treatment—Where do we Stand? A State of the Art Review,” Cancer Treatment Reviews 40, no. 4 (2014): 523–532. 24345772 10.1016/j.ctrv.2013.11.006

[4] I. Panez‐Toro , J. Munoz‐Garcia , J. W. Vargas‐Franco , et al., “Advances in Osteosarcoma,” Current Osteoporosis Reports 21, no. 4 (2023): 330–343. 37329384 10.1007/s11914-023-00803-9 PMC10393907

[5] S. Wang , Q. Ren , G. Li , X. Zhao , X. Zhao , and Z. Zhang , “The Targeted Therapies for Osteosarcoma via Six Major Pathways,” Current Molecular Pharmacology 17, no. 1 (2024): e369822605. 10.2174/1874467217666230821142839 37602543

[6] R. B. Damgaard , “The Ubiquitin System: From Cell Signalling to Disease Biology and New Therapeutic Opportunities,” Cell Death and Differentiation 28, no. 2 (2021): 423–426. 33446876 10.1038/s41418-020-00703-w PMC7862391

[7] T. Sun , Z. Liu , and Q. Yang , “The Role of Ubiquitination and Deubiquitination in Cancer Metabolism,” Molecular Cancer 19, no. 1 (2020): 146. 33004065 10.1186/s12943-020-01262-x PMC7529510

[8] M. J. Zhou , F. Z. Chen , and H. C. Chen , “Ubiquitination Involved Enzymes and Cancer,” Medical Oncology 31, no. 8 (2014): 93. 25023052 10.1007/s12032-014-0093-6

[9] S. Fang , K. L. Lorick , J. P. Jensen , and A. M. Weissman , “RING Finger Ubiquitin Protein Ligases: Implications for Tumorigenesis, Metastasis and for Molecular Targets in Cancer,” Seminars in Cancer Biology 13, no. 1 (2003): 5–14. 12507552 10.1016/s1044-579x(02)00095-0

[10] C. T. Chasapis and G. A. Spyroulias , “RING Finger E(3) ubiquitin Ligases: Structure and Drug Discovery,” Current Pharmaceutical Design 15, no. 31 (2009): 3716–3731. 19925422 10.2174/138161209789271825

[11] H. Oshiumi , M. Matsumoto , S. Hatakeyama , and T. Seya , “Riplet/RNF135, a RING Finger Protein, Ubiquitinates RIG‐I to Promote Interferon‐Beta Induction During the Early Phase of Viral Infection,” Journal of Biological Chemistry 284, no. 2 (2009): 807–817. 19017631 10.1074/jbc.M804259200

[12] Y. Yao , G. Gong , Z. Guo , and D. Zhang , “A pan‐Cancer Analysis of Ring Finger Protein 135 and Its Relationship to Triple‐Negative Breast Cancer Proliferation and Metastasis,” Aging 14, no. 23 (2022): 9758–9772. 36495591 10.18632/aging.204429 PMC9792201

[13] J. Jin , L. Zhao , and Z. Li , “The E3 Ubiquitin Ligase RNF135 Regulates the Tumorigenesis Activity of Tongue Cancer SCC25 Cells,” Cancer Medicine 5, no. 11 (2016): 3140–3146. 27709798 10.1002/cam4.832 PMC5119969

[14] M. Alizada , J. Li , H. Aslami , D. Yang , T. Korchuganova , and Y. H. Xu , “Beta‐Elemene Inhibits the Proliferation and Migration of Human Glioblastoma Cell Lines via Suppressing Ring Finger Protein 135,” Balkan Journal of Medical Genetics: BJMG 23, no. 1 (2020): 43–49. 32953408 10.2478/bjmg-2020-0002 PMC7474225

[15] Y. Liu , F. Wang , Y. Liu , et al., “RNF135, RING Finger Protein, Promotes the Proliferation of Human Glioblastoma Cells in Vivo and in Vitro via the ERK Pathway,” Scientific Reports 6 (2016): 20642. 26856755 10.1038/srep20642 PMC4746631

[16] X. Wang , M. Chen , X. Liang , et al., “RNF135 Promoter Methylation is Associated With Immune Infiltration and Prognosis in Hepatocellular Carcinoma,” Frontiers in Oncology 11 (2021): 752511. 35145901 10.3389/fonc.2021.752511 PMC8821516

[17] G. Guan , Y. Zhang , Y. Lu , et al., “The HIF‐1alpha/CXCR4 Pathway Supports Hypoxia‐Induced Metastasis of Human Osteosarcoma Cells,” Cancer Letters 357, no. 1 (2015): 254–264. 25444927 10.1016/j.canlet.2014.11.034

[18] K. Shi , S. L. Wang , B. Shen , F. Q. Yu , D. F. Weng , and J. H. Lin , “Clinicopathological and Prognostic Values of Fibronectin and Integrin αvβ3 Expression in Primary Osteosarcoma,” World Journal of Surgical Oncology 17, no. 1 (2019): 23. 30691475 10.1186/s12957-019-1566-z PMC6350278

[19] S. A. Lakhani , A. Masud , K. Kuida , et al., “Caspases 3 and 7: Key Mediators of Mitochondrial Events of Apoptosis,” Science 311, no. 5762 (2006): 847–851. 16469926 10.1126/science.1115035 PMC3738210

[20] J. Song , X. Yuan , L. Piao , et al., “Cellular Functions and Molecular Mechanisms of Ubiquitination in Osteosarcoma,” Frontiers in Oncology 12 (2022): 1072701. 36530999 10.3389/fonc.2022.1072701 PMC9753703

[21] O. Egorova , H. H. Lau , K. McGraphery , and Y. Sheng , “Mdm2 and MdmX RING Domains Play Distinct Roles in the Regulation of p53 Responses: A Comparative Study of Mdm2 and MdmX RING Domains in U2OS Cells,” International Journal of Molecular Sciences 21, no. 4 (2020): 1309. 32075226 10.3390/ijms21041309 PMC7072982

[22] X. Guan , Y. Xu , and J. Zheng , “Long Non‐Coding RNA PCAT6 Promotes the Development of Osteosarcoma by Increasing MDM2 Expression,” Oncology Reports 44, no. 6 (2020): 2465–2474. 33125146 10.3892/or.2020.7813 PMC7610325

[23] Z. Wang , X. Xu , W. Tang , Y. Zhu , J. Hu , and X. Zhang , “Tripartite Motif Containing 11 Interacts With DUSP6 to Promote the Growth of Human Osteosarcoma Cells Through Regulating ERK1/2 Pathway,” BioMed Research International 2019 (2019): 9612125. 31950060 10.1155/2019/9612125 PMC6948331

[24] W. Jiang , X. Cai , T. Xu , et al., “Tripartite Motif‐Containing 46 Promotes Viability and Inhibits Apoptosis of Osteosarcoma Cells by Activating NF‐B Signaling Through Ubiquitination of PPAR,” Oncology Research 28, no. 4 (2020): 409–421. 32295675 10.3727/096504020X15868639303417 PMC7851538

[25] C. Zhou , Z. Zhang , X. Zhu , et al., “N6‐Methyladenosine Modification of the TRIM7 Positively Regulates Tumorigenesis and Chemoresistance in Osteosarcoma Through Ubiquitination of BRMS1,” eBioMedicine 59 (2020): 102955. 32853985 10.1016/j.ebiom.2020.102955 PMC7452680

[26] J. W. Harper and B. A. Schulman , “Cullin‐RING Ubiquitin Ligase Regulatory Circuits: A Quarter Century Beyond the F‐Box Hypothesis,” Annual Review of Biochemistry 90 (2021): 403–429. 10.1146/annurev-biochem-090120-013613 PMC8217159 33823649

[27] Z. Chen , W. Zhang , K. Jiang , et al., “MicroRNA‐300 Regulates the Ubiquitination of PTEN Through the CRL4B(DCAF13) E3 Ligase in Osteosarcoma Cells,” Molecular Therapy–Nucleic Acids 10 (2018): 254–268. 29499938 10.1016/j.omtn.2017.12.010 PMC5768150

[28] B. Chen , Y. Feng , M. Zhang , G. Cheng , B. Chen , and H. Wang , “Small Molecule TSC01682 Inhibits Osteosarcoma Cell Growth by Specifically Disrupting the CUL4B‐DDB1 Interaction and Decreasing the Ubiquitination of CRL4B E3 Ligase Substrates,” American Journal of Cancer Research 9, no. 9 (2019): 1857–1870. 31598391 PMC6780663

[29] P. Yuan , Y. Zhou , R. Wang , et al., “TRIM58 Interacts With Pyruvate Kinase M2 to Inhibit Tumorigenicity in Human Osteosarcoma Cells,” BioMed Research International 2020 (2020): 8450606. 32219144 10.1155/2020/8450606 PMC7081029

[30] Q. Zhao , N. Liu , T. Xu , and K. Song , “RING Finger Gene 180 Inhibits Osteosarcoma Progression Through Regulating Chromobox Homolog 4 Ubiquitination,” Cell Cycle 22, no. 10 (2023): 1246–1258. 37095741 10.1080/15384101.2023.2205201 PMC10193903

[31] Z. Bai , C. Yao , J. Zhu , et al., “Anti‐Tumor Drug Discovery Based on Natural Product Beta‐Elemene: Anti‐Tumor Mechanisms and Structural Modification,” Molecules 26, no. 6 (2021): 1499. 33801899 10.3390/molecules26061499 PMC7998186

[32] Z. Q. Wang , M. Y. Zhang , M. L. Deng , N. Q. Weng , H. Y. Wang , and S. X. Wu , “Low Serum Level of miR‐485‐3p Predicts Poor Survival in Patients With Glioblastoma,” PLoS One 12, no. 9 (2017): e0184969. 28931080 10.1371/journal.pone.0184969 PMC5607158

[33] Y. Zhang , R. Sui , Y. Chen , H. Liang , J. Shi , and H. Piao , “Downregulation of miR‐485‐3p Promotes Glioblastoma Cell Proliferation and Migration via Targeting RNF135,” Experimental and Therapeutic Medicine 18, no. 1 (2019): 475–482. 31258684 10.3892/etm.2019.7600 PMC6566029

[34] E. Pasmant , J. Masliah‐Planchon , P. Levy , et al., “Identification of Genes Potentially Involved in the Increased Risk of Malignancy in NF1‐Microdeleted Patients,” Molecular Medicine 17, no. 1–2 (2011): 79–87. 20844836 10.2119/molmed.2010.00079 PMC3022985

[35] L. Guo , D. Kong , J. Liu , et al., “Breast Cancer Heterogeneity and Its Implication in Personalized Precision Therapy,” Experimental Hematology & Oncology 12, no. 1 (2023): 3. 36624542 10.1186/s40164-022-00363-1 PMC9830930

[36] A. A. Alizadeh , V. Aranda , A. Bardelli , et al., “Toward Understanding and Exploiting Tumor Heterogeneity,” Nature Medicine 21, no. 8 (2015): 846–853. 10.1038/nm.3915 PMC4785013 26248267

[37] M. R. Khezri , R. Jafari , K. Yousefi , and N. M. Zolbanin , “The PI3K/AKT Signaling Pathway in Cancer: Molecular Mechanisms and Possible Therapeutic Interventions,” Experimental and Molecular Pathology 127 (2022): 104787. 35644245 10.1016/j.yexmp.2022.104787

[38] P. Wolf , “Inhibitor of Apoptosis Proteins as Therapeutic Targets in Bladder Cancer,” Frontiers in Oncology 13 (2023): 1124600. 36845731 10.3389/fonc.2023.1124600 PMC9950391

