# Supplementary figures and images for: RNF135 Promotes Human Osteosarcoma Cell Growth and Inhibits Apoptosis by Upregulating the PI3K/AKT Pathway
Source: Cancer Rep (Hoboken). 2024 Aug 8;7(8):e2159. doi: 10.1002/cnr2.2159 (PMC11310095; doi:10.1002/cnr2.2159)

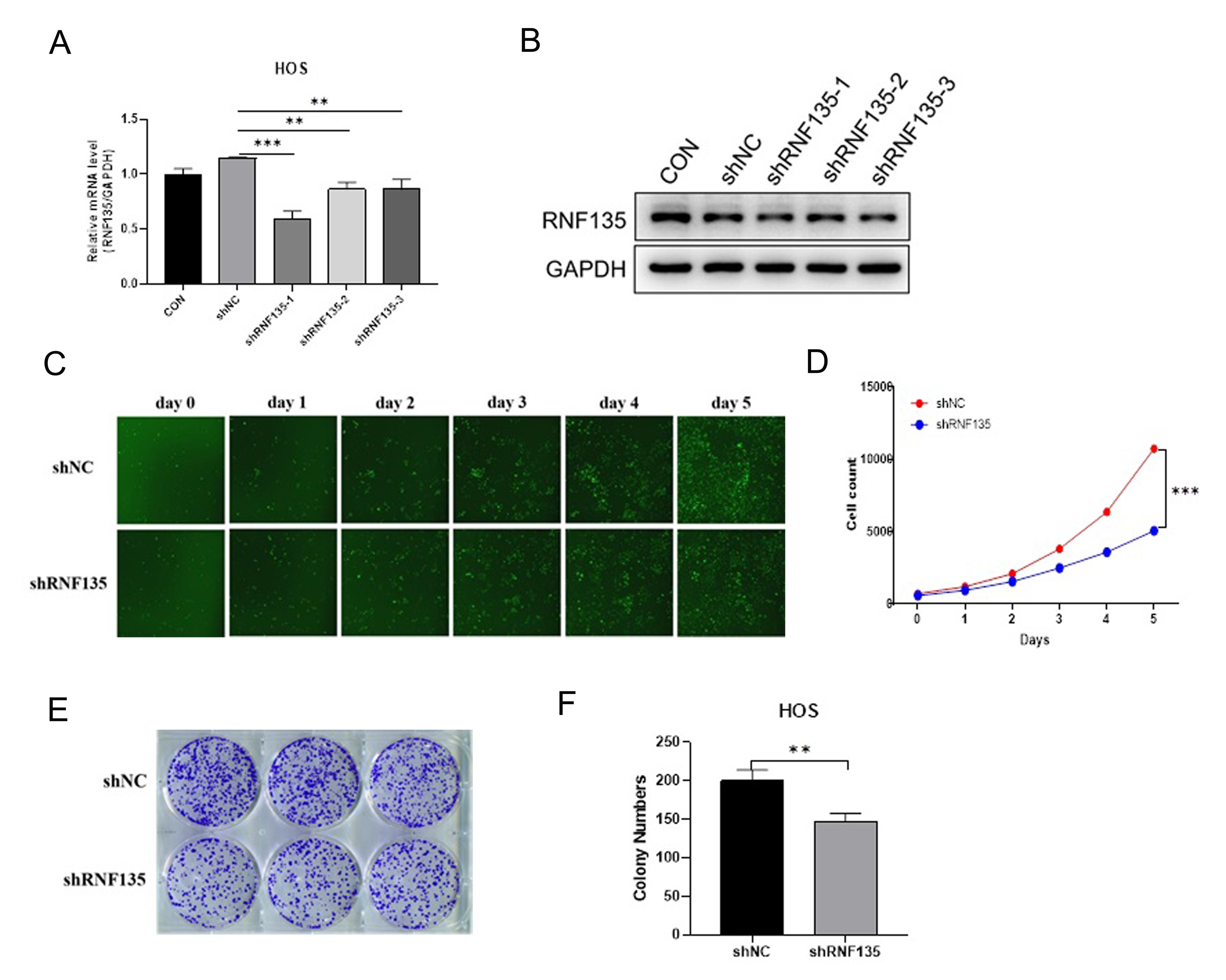

Supplement: Supplementary file 1 — Figure S1. RNF135 knockdown inhibits HOS cell proliferation and colony formation. (A, B) HOS cells were transfected with shRNF135‐1, ‐2, ‐3, or shNC. RNF135 mRNA (A) and protein (B) levels were determined by qRT‐PCR and western blot analysis, respectively. n = 3, **p < 0.01, ***p < 0.001. (C, D) Stable RNF135 knockdown and shNC control HOS cells were cultured in 96‐well plates for 5 days. Representative fluorescence cell images (C) and cell growth curve (D) are shown. n = 3, ***p < 0.001. (E, F) Stable RNF135 knockdown and shNC control HOS cells were cultured in 6‐wells plates for 15 days. Representative cell images (E) and the number of colonies formed (F) on day 15 are shown. n = 3, **p < 0.01. [file CNR2-7-e2159-s001.jpg]

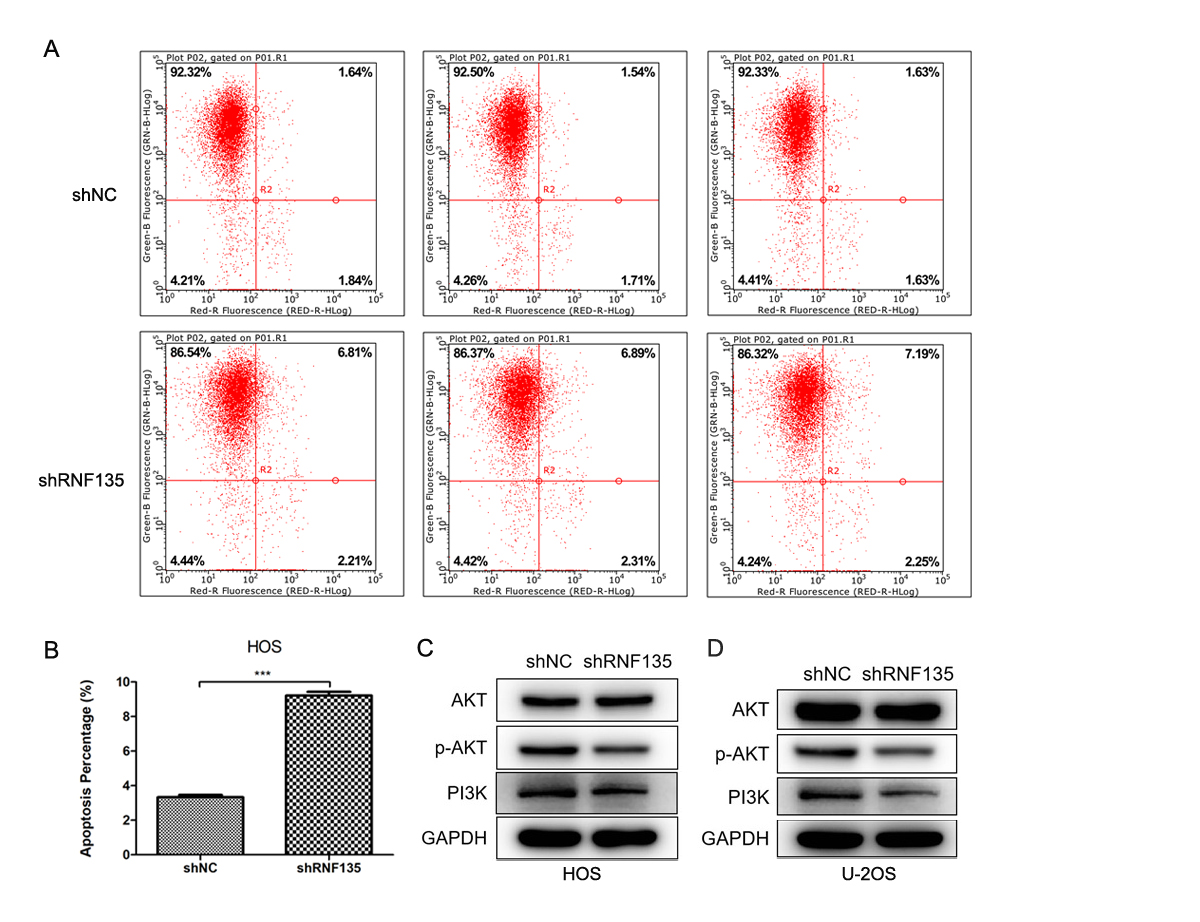

Supplement: Supplementary file 2 — Figure S2. RNF135 knockdown downregulates PI3K/AKT and induces HOS cell apoptosis. Stable RNF135 knockdown HOS and U2OS cells and their respective shNC control cells were cultured in 6‐well plates for 24 h. (A, B) Flow cytometric analysis of HOS cell apoptosis using Annexin V staining. Representative flow cytometry histograms (A) and quantified apoptotic cell percentages (B) are shown. n = 3, ***p < 0.001. (C, D) The protein levels of AKT, p‐AKT, and PI3K in HOS (C) and U2OS (D) cells determined by western blot analysis. [file CNR2-7-e2159-s002.jpg]
